# CONTEXTUALIZING ATTITUDES TOWARD MEDICAL AID IN DYING IN A NATIONAL SAMPLE OF HOSPICE CLINICIANS

**DOI:** 10.1093/geroni/igad104.3700

**Published:** 2023-12-21

**Authors:** Todd Becker, John Cagle, Joan Davitt, Paul Sacco, Nancy Kusmaul, Cindy Cain

**Affiliations:** Washington University in St. Louis, St. Louis, Missouri, United States; University of Maryland, Baltimore, Baltimore, Maryland, United States; University of Maryland, Baltimore, Baltimore, Maryland, United States; University of Maryland, Baltimore, Baltimore, Maryland, United States; University of Maryland Baltimore County, Baltimore, Maryland, United States; University of Alabama at Birmingham, Birmingham, Alabama, United States

## Abstract

Research on the contextual characteristics informing the care that hospice professionals offer patients interested in medical aid in dying (MAID) is lacking. Thus, we aimed to explore MAID attitudes vis-à-vis hospice values and state law. This cross-sectional study recruited a convenience sample of 450 hospice-based physicians, nurses, social workers, and chaplains from national hospice and palliative care membership associations. Controlling for covariates, we fit a partial proportional odds model with clustered standard errors to examine the associations between participants’ professional experience working in a MAID-legal state, orientation toward patient-centered care, and commitment to the hospice philosophy of care, and their MAID attitudes (0 = “oppose,” 1 = “neither support nor oppose,” 2 = “support”). Results indicated that experience working in MAID-legal states (vs. not; OR = 2.42, p = .002) and increased orientation toward patient-centered care (OR = 1.38, p < .001) were significantly associated with higher odds of greater MAID support across thresholds. Conversely, the direction and statistical significance of commitment to the hospice philosophy of care depended on the threshold of the dependent variable (odds of MAID support higher than “oppose”: OR = 0.98, p = .326; odds of MAID support higher than “neither support nor oppose”: OR = 1.05, p = .043). Findings provide the first quantitative evaluation of a predominantly theoretical debate concerning perceptions of MAID’s concordance with hospice values. Noting legal protections for conscientious objection, higher support from clinicians in states where MAID is legal may suggest training opportunities as levers to improve patient access.

